# Combined Respiratory Chain Deficiency and *UQCC2* Mutations in Neonatal Encephalomyopathy: Defective Supercomplex Assembly in Complex III Deficiencies

**DOI:** 10.1155/2017/7202589

**Published:** 2017-07-19

**Authors:** René G. Feichtinger, Michaela Brunner-Krainz, Bader Alhaddad, Saskia B. Wortmann, Reka Kovacs-Nagy, Tatjana Stojakovic, Wolfgang Erwa, Bernhard Resch, Werner Windischhofer, Sarah Verheyen, Sabine Uhrig, Christian Windpassinger, Felix Sternberg, Christine Makowski, Tim M. Strom, Thomas Meitinger, Holger Prokisch, Wolfgang Sperl, Tobias B. Haack, Johannes A. Mayr

**Affiliations:** ^1^Department of Pediatrics, Salzburger Landeskliniken (SALK) and Paracelsus Medical University (PMU), 5020 Salzburg, Austria; ^2^Division of General Pediatrics, Department of Pediatrics and Adolescent Medicine, Medical University of Graz, 8010 Graz, Austria; ^3^Institute of Human Genetics, Technische Universität München, 81675 Munich, Germany; ^4^Institute of Human Genetics, Helmholtz Zentrum München, 85764 Neuherberg, Germany; ^5^Clinical Institute of Medical and Chemical Laboratory Diagnostics, Medical University of Graz, 8010 Graz, Austria; ^6^Division of Neonatology, Department of Pediatrics and Adolescent Medicine, Medical University of Graz, 8010 Graz, Austria; ^7^Department of Human Genetics, Medical University of Graz, 8010 Graz, Austria; ^8^Laura Bassi Centre of Expertise-THERAPEP, Research Program for Receptor Biochemistry and Tumor Metabolism, Department of Pediatrics, Paracelsus Medical University, 5020 Salzburg, Austria; ^9^Department for Paediatric and Adolescent Medicine, Schwabing Hospital, Technische Universität München, 80804 Munich, Germany

## Abstract

Vertebrate respiratory chain complex III consists of eleven subunits. Mutations in five subunits either mitochondrial (MT-CYB) or nuclear (CYC1, UQCRC2, UQCRB, and UQCRQ) encoded have been reported. Defects in five further factors for assembly (TTC19, UQCC2, and UQCC3) or iron-sulphur cluster loading (BCS1L and LYRM7) cause complex III deficiency. Here, we report a second patient with UQCC2 deficiency. This girl was born prematurely; pregnancy was complicated by intrauterine growth retardation and oligohydramnios. She presented with respiratory distress syndrome, developed epileptic seizures progressing to status epilepticus, and died at day 33. She had profound lactic acidosis and elevated urinary pyruvate. Exome sequencing revealed two homozygous missense variants in *UQCC2*, leading to a severe reduction of UQCC2 protein. Deficiency of complexes I and III was found enzymatically and on the protein level. A review of the literature on genetically distinct complex III defects revealed that, except TTC19 deficiency, the biochemical pattern was very often a combined respiratory chain deficiency. Besides complex III, typically, complex I was decreased, in some cases complex IV. In accordance with previous observations, the presence of assembled complex III is required for the stability or assembly of complexes I and IV, which might be related to respirasome/supercomplex formation.

## 1. Introduction

Vertebrate complex III (coenzyme Q:cytochrome c oxidoreductase) consists of 11 subunits. Mutations in five subunits (MT-CYB, CYC1, UQCRC2, UQCRB, and UQCRQ), three factors for protein complex assembly (TTC19, UQCC2, and UQCC3), and two factors (BCS1L and LYRM7) involved in loading of the [2Fe-2S] iron sulphur cofactor on the Rieske protein of complex III were described [1–10]. *MT-CYB* is a mitochondrial gene-encoding cytochrome *b*. Complex III transports electrons from ubiquinol to cytochrome *c*. Cytochrome *c*1, cytochrome *b*, and the Rieske protein represent the redox center. The heme group from cytochrome *c*1 is located in the intermembrane space, where it accepts electrons from the Rieske protein. Complex III is associated with complexes I and IV to form the respirasome. UQCC2 is required for complex III assembly. The protein can affect insulin secretion, mitochondrial ATP production, and myogenesis via modulation of the respiratory chain activity [11]. UQCC2 interacts with UQCC1 to mediate cytochrome *b* expression and subsequent complex III assembly [7]. UQCC1 and 2 might specifically bind to newly synthesized cytochrome *b* at the nucleotide where they are stabilized [7, 12]. UQCC2 is mainly expressed in the brain, kidney, heart, and skeletal muscle [12].

The majority of complex I is found bound with a complex III dimer and complex IV (CI, CIII_2_, and CIV) termed respirasome or with a complex III dimer alone (CI, CIII_2_). In addition, CIII dimers can form a complex with CIV (CIII_2_CIV) independent of complex I. Very recently, the structure of respiratory chain supercomplexes revealed several interaction sites between complexes III, I, and IV. In total, nine interaction sites between supercomplexes were described [13].

All complex III deficiencies show an autosomal recessive mode of inheritance with the exception of cytochrome *b* defects that either show maternal inheritance or occur spontaneously, since this subunit is encoded by the mitochondrial genome (mtDNA). Heterogeneous clinical phenotypes have been described in relation to respiratory chain complex III deficiency.

Here, we report on a second patient with mutations in the complex III assembly factor *UQCC2* who showed pronounced deficiency of complexes I and III. Furthermore, we give an overview on the biochemical findings in patients with mutations in distinct complex III subunits or assembly factors.

## 2. Material and Methods

### 2.1. Ethics

The study was performed according to the Austrian Gene Technology Act. Experiments were conducted in accordance with the Helsinki Declaration of 1975 (revised 1983) and the guidelines of the Salzburg State Ethics Research Committee (ethical agreement: AZ 209-11-E1/823-2006), being no clinical drug trial or epidemiological investigation. All clinical data and samples were obtained with written informed consent of the patients' parents. The ethical committee of the Technische Universität München approved the exome sequencing studies.

### 2.2. Exome Sequencing

Exome sequencing was performed from peripheral-blood DNA samples as reported previously [14]. In brief, coding regions were enriched using a SureSelect Human All Exon V5 kit (Agilent) followed by sequencing as 100 base-pairs paired-end runs on an Illumina HiSeq2500. Reads were aligned to the human reference genome (UCSC Genome Browser build hg19) using Burrows-Wheeler Aligner (v.0.7.5a) [15]. Single-nucleotide variants and small insertions and deletions (indels) were detected with SAMtools (version 0.1.19) [16].

Confirmation was performed by Sanger sequencing using the following forward 5′5-CTCCCGCTCCACTCCTAAG-3′ and reverse 5′-GTCCTTTCCTCCCCTCGTC-3′ primers.

### 2.3. Enzyme Activity of the OXPHOS Complexes

Spectrophotometric measurement of OXPHOS enzyme and citrate synthase activity was performed as previously described [17, 18]. Muscle tissue (20–100 mg) was homogenized in extraction buffer (250 mM sucrose, 40 mM KCl, 2 mM EGTA, 20 mM Tris-HCl, pH 7.6). The postnuclear supernatant (600 ×g homogenate) containing the mitochondrial fraction was used for measurement of enzyme activities and Western blot analysis.

### 2.4. Western Blot Analysis on SDS-PAGE

600*g* homogenates were separated on acrylamide/bisacrylamide gels and transferred to nitrocellulose membranes. Immunological detection of proteins was carried out as described previously [17]. The following primary antibodies were used: polyclonal rabbit anti-UQCC2 (Proteintech, 1 : 1000, overnight 4°C), monoclonal mouse anti-NDUFS4 (Abcam, 1 : 1000, 1 h RT), monoclonal mouse anti-UQCRC2 (Abcam, 1 : 1500, 1 h RT), polyclonal rabbit anti-COX2 (Abcam, 1 : 1000, overnight 4°C), monoclonal mouse anti-porin 31HL (Abcam, 1 : 1000, 1 h RT), and polyclonal rabbit anti-GAPDH (Trevigen, 1 : 5000, 1 h RT). GAPDH was used as a loading control.

### 2.5. Blue Native-PAGE (Lauryl Maltoside Solubilization)

For blue-native gel electrophoresis, 600 ×g supernatants of muscle tissue or isolated mitochondria from fibroblasts were used. Solubilized mitochondrial membranes were prepared from isolated fibroblast mitochondria as described previously [19]. Briefly, fibroblast mitochondria or 600*g* supernatants were sedimented by centrifugation at 13,000*g* for 15 min. Samples were solubilized with 1.5% lauryl maltoside for 15 min and centrifuged for 20 min at 13,000*g*. Samples were loaded on a 5% to 13% polyacrylamide gradient gel and separated electrophoretically. For immunoblot analysis, preparations were separated by BN-PAGE (5–13%) and blotted onto polyvinylidene difluoride membrane (Hybond-P, GE Healthcare) using a CAPS buffer (10 mmol/l 3-cyclohexylamino-1-propane sulfonic acid pH 11, 10% methanol). The membrane was washed in 100% methanol for 2 min and blocked for 30 min at room temperature in 1% blocking solution (Roche) dissolved in TBS-T. The primary antibodies, diluted in 1% blocking solution-TBS-T, were added 1 h at room temperature. The following primary antibody dilutions were used: complex I subunit NDUFS4 monoclonal antibody (1 : 1000; Abcam), complex III subunit core 2 monoclonal antibody (1 : 1500; Abcam), and complex V subunit *α* monoclonal antibody (1 : 1000; Abcam). After extensive washing, blots were incubated for 1 h at RT with secondary mouse antibody (1 : 100; DAKO polymer Envision Staining Kit). Detection was carried out with Lumi-LightPLUS POD substrate (Roche).

### 2.6. Blue Native-PAGE (Digitonin Solubilization)

Gel electrophoresis was performed as previously described [20]. The blotting procedure is described in Section 2.5. The following primary antibody dilutions were used: complex I subunit NDUFS4 monoclonal antibody (1 : 1000; Abcam), complex V subunit *α* monoclonal antibody (1 : 1000; Abcam), complex III subunit Core 2 monoclonal antibody (1 : 1500; Abcam), complex IV subunit 2 polyclonal antibody (1 : 1000; Abcam), and complex II subunit SDHA monoclonal antibody (1 : 30,000; Abcam). The PVDF membrane was incubated with COX2 and NDUFS4 antibodies overnight at 4°C. Incubation with all other primary antibodies was performed for 1 h at RT.

### 2.7. Immunofluorescence Staining

Fibroblasts were grown on chamber slides. Cells were allowed to attach for 24 hours. At the next day, the medium was removed, and chamber slides were twice washed with PBS pH 7.4 and fixated in formalin overnight at 4°C. After washing cells three times 3 min with PBS-T (pH 7.5; 0.05% Tween-20), heat-induced epitope retrieval was done in 1 mM EDTA, 0.01% Tween-20, pH 8 at 95°C for 45 min. The solution was allowed to cool down to room temperature and chamber slides were washed with PBS-T. The chamber slides were incubated 1 h at RT with primary antibodies against rabbit-polyclonal porin 31HL (1 : 400), mouse-monoclonal NDUFS4 (1 : 100), and mouse-monoclonal UQCRC2 (1 : 400). 1st antibodies were diluted in DAKO antibody diluent with background-reducing components. After washing with PBS-T, cells were incubated 1 h at RT in dark with secondary antibodies (Alexa Fluor 594 donkey anti-rabbit antibody, 1 : 500 and Alexa Fluor 488 donkey anti-mouse IgG (H + L), 1 : 1000). After washing the chamber slides with PBS-T, they were incubated with DAPI diluted 1 : 2000 in PBS-T for 10 min. Chamber slides were mounted in fluorescence mounting media from DAKO.

## 3. Results

### 3.1. Clinical Report

The girl is the first child of healthy consanguineous Turkish parents (first-degree cousins). Pregnancy was complicated by intrauterine growth restriction (IUGR), oligohydramnios, and breech presentation. She was born at 32 weeks of gestation by Caesarean section, body weight: 1430 g (<25th centile), body length: 42 cm (3rd centile), and head circumference: 30.7 cm (10th centile). APGAR scores were 8/9/9 after 1/5/10 minutes, and umbilical artery pH was 7.34.

She suffered from respiratory distress syndrome (IRDS) grades III-IV and required CPAP-ventilation followed by endotracheal intubation and mechanical ventilation at the age of three hours. Within the first 4 days of life, she required 4 times surfactant (Curosurf©) replacement therapy. At day 14, she presented with pulmonary haemorrhage and the first epileptic seizures which progressed into a status epilepticus. Despite total parenteral nutrition (oral feedings caused recurrent vomiting), weight gain remained poor. She had no obvious dysmorphic features, physical examination was unremarkable, and neurological examination was influenced by sedation and analgesia during continuous mechanical ventilation, but showed a generally low muscle tone. Ophthalmological investigation was normal, a hearing test was not performed, and echocardiography and ECG revealed normal results. The EEG showed low-voltage activity. Repetitive cranial ultrasound examinations showed periventricular echodensities, a noncalcifying vasculopathy in the basal ganglia region, and signs of general hypoxemic encephalopathy. She died due to respiratory failure on day 33.

### 3.2. Laboratory Findings

A profound and recurrent lactic acidosis (max. 20 mmol/l, ref. <2.4 mmol/l) was evident starting at the age of 4 hours and lasting until her death. Extended newborn screening was unremarkable. Blood counts, C-reactive protein, interleukin-6, procalcitonin, and liver enzymes were repetitively within normal limits, creatinine (1.49 mg/dl) was slightly elevated (ref. 0.90–1.40 mg/dl), urea was normal (39 mg/dl), and coagulation analysis was within normal limits. High pyruvate excretion was noted. The karyotyping (46 XX) was unremarkable. SNP analysis confirmed consanguinity of the patients' parents and revealed a large homozygosity-by-descent (HBD) region of about 54 Mb including almost the entire p-arm of chromosome 6; this area contains 690 genes, including *UQCC2* as potential disease-causing gene.

### 3.3. Exome Sequencing

**Figure 1:**
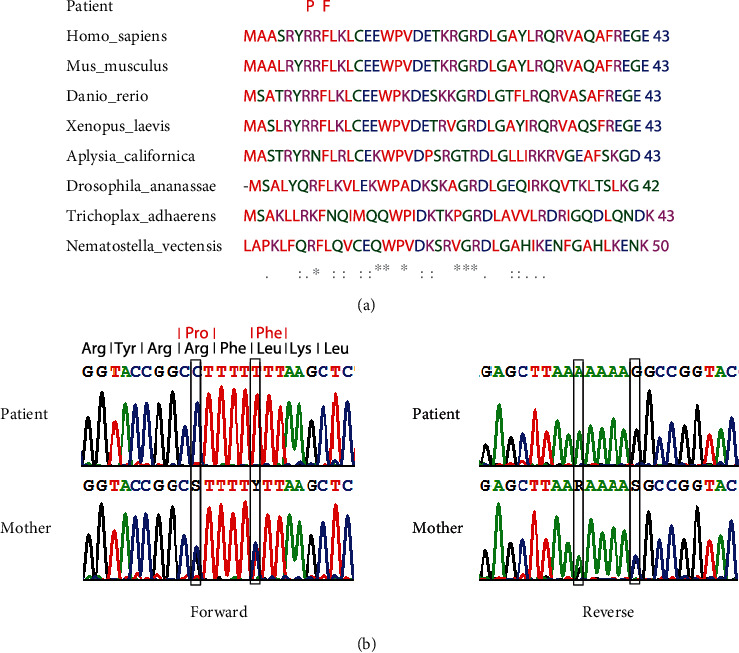
Conservation of affected amino acid residues and enzymatic activities of respiratory chain complexes in the muscle and fibroblasts. (a) Phylogenetic conservation of UQCC2. The two homozygous missense mutation affect highly conserved amino acid residues: c.[23G>C; 28C>T]; [23G>C; 28C>T], (p.[Arg8Pro; Leu10Phe]; [Arg8Pro; Leu10Phe]), and reference sequence GenBank NM_032340.3. (b) UQCC2-sequencing chromatograms of the patient and mother.

Sequencing revealed two homozygous missense mutations c.[23G>C;28C>T];[23G>C;28C>T] and p.[Arg8Pro;Leu10Phe];[Arg8Pro;Leu10Phe] in the ubiquinol-cytochrome c reductase complex assembly factor 2, encoded by *UQCC2*, GenBank accession NM_032340.3 (Figure 1). Both mutations affect phylogenetically conserved amino acids and were predicted to be of pathogenic relevance by all used prediction programs (Polyphen-2, SIFT, Provean, MutationTaster, CADD; Supplemental Table 1 available online at https://doi.org/10.1155/2017/7202589). Both variants seem to be very rare since they are neither found in 1000 Genome nor in the ExAC databases. These variants were confirmed by the Sanger sequencing; the mother is a heterozygous carrier (Figure 1(b)).

### 3.4. Enzymatic Measurements

Spectrophotometric measurement revealed a combined reduction of complex III (111 mUnits/mg protein; normal range: 230–486) and complex I (9 mUnits/mg protein; normal range: 30–84) in the muscle (Table 1). No reduction of respiratory chain enzymes was obvious in patient fibroblasts (Table 1) suggesting some tissue specificity of the UQCC2 defect.

### 3.5. Western Blot Analysis

**Figure 2:**
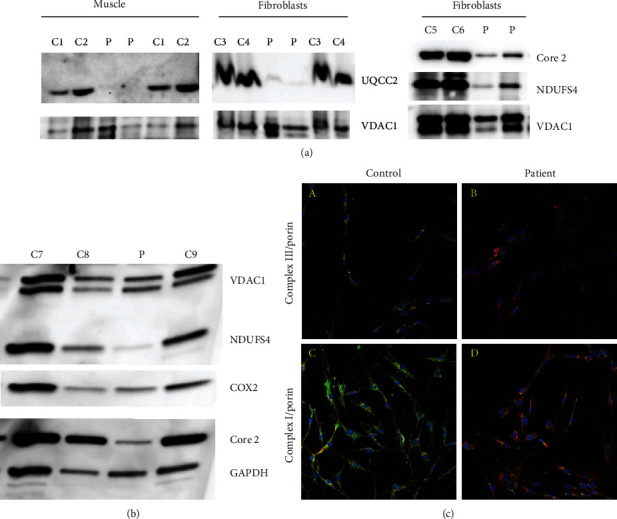
SDS Western blot analysis of muscle and immunofluorescence staining of the OXPHOS complexes of fibroblasts. (a) SDS Western blot analysis of UQCC2, Core 2, NDUFS4, and VDAC1 of muscle 600 g supernatant and isolated fibroblast mitochondria. Two different amounts of mitochondrial proteins were loaded of controls and patient muscle/fibroblasts. (b) SDS Western blot analysis of the OXPHOS complexes of muscle 600 g supernatant of the OXPHOS complexes. (c) Immunofluorescence staining of complexes III and I in fibroblasts. (A, B) Staining of complex III and porin. (C, D) Staining of complex I and porin. (A, C) Control. (B, D) Patient. Magnification 20x.

Almost complete loss of UQCC2 protein was present in the muscle and fibroblasts of the affected individual (Figure 2(a)). A severe reduction of complex III and complex I in the muscle and fibroblasts was also confirmed by the immunoblot analyzed (Figures 2(a) and 2(b)). Complex IV was not affected (Figure 2(b)).

### 3.6. Immunofluorescence Staining

A reduction of protein amount of both complex III and complex I was present in patient fibroblasts as found by immunohistochemical staining (Figure 2(c)).

### 3.7. Blue-Native Gel Electrophoresis

**Figure 3:**
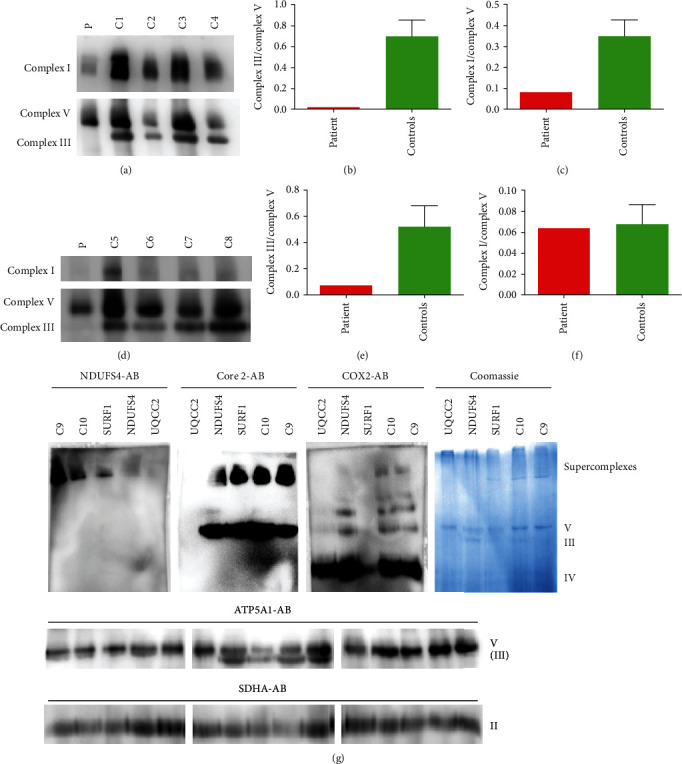
Blue native (BN) gel electrophoresis of muscle and fibroblasts. (a) BN-PAGE of the muscle with lauryl maltoside solubilization. (b, c) Densitometric analysis of BN-PAGE from the muscle. (d) BN-PAGE of fibroblasts. (e, f) Densitometric analysis of BN-PAGE from fibroblasts. (g) BN-PAGE of the muscle with a digitonin solubilization. The membrane was incubated with antibodies targeted against NDUFS4, Core 2, COX2, ATP5A1, and SDHA. C1–C10 are normal controls. In addition, muscle homogenates from patients with loss of function mutations in either *SURF1* (NM_003172.3) c.11_30del20 (p.Val4Alafs^∗^49) or *NDUFS4* (NM_002495.2) c.466_469dupAAGT (p.Ser157^∗^) were used as disease controls.

A decreased amount of assembled complexes III and I was present in the muscle (Figure 3(a)). In fibroblasts, complex III was decreased as well, whereas normal amounts of complex I were detected (Figure 3(d)). Loss of complex III and supercomplexes containing complex III was observed in digitonin solubilized muscle of the UQCC2 patient as shown by Western blot analysis with an AB raised against the core 2 subunit of complex III and staining with Serva Blue G (Figure 3(g)). A reduction was also present in a patient with a pathogenic NDUFS4 mutation. No significant reduction was present in the muscle of a patient carrying a pathogenic SURF1 mutation. To further underline the findings, the Western blot was also analyzed with antibodies against NDUFS4 and COX2. A reduction of complex I containing supercomplexes was observed in muscle homogenates of the UQCC2-deficient patient and also in patients with loss of function mutations in either NDUFS4 or SURF1 (Figure 3(g)) shown with an antibody against NDUFS4. No signal was present at the size of the supercomplexes for COX2 in the muscle of all patients compared to healthy controls. Normal amounts of monomeric complex IV were present in UQCC2 and NDUFS4 muscle, whereas a severe reduction was found in the SURF1 patient. No differences were present regarding complexes V and II between patients and controls.

## 4. Discussion

Here, we report on a second patient with mutations in *UQCC2*, an assembly factor of complex III. The clinical phenotype in our patient is difficult to interpret as many findings seem to be related to the prematurity (IRDS with respiratory problems and periventricular echodensities on brain ultrasound). However, as she was born at a gestational age of 32 weeks, the clinical course was clearly more severe than expected usually for preterm infants at this week of gestation: key features represented the recurrent neonatal seizures and severe lactic acidosis. These findings might indicate that the severe course of the disease was due to the underlying mitochondrial disorder. Further speculation about a distinctive clinical pattern is impossible due to mechanical ventilation throughout her life and the influence of sedation and analgesia. Interestingly, clinical information on the first reported patient was also limited [7]. It might be worth mentioning that IUGR was found in our patient and also in the other UQCC2 patient, while this has not been reported for other complex III-related genetic disorders.

In general, the clinical course in relation with respiratory chain complex III deficiency is heterogenous [21]. Given the small number of patients reported, it might be even broader (Table 2). Based on clinical presentation, it is impossible to distinguish between subunits or assembly factor defects.

A distinctive and comparable clinical pattern was reported in six patients from five families. These individuals with mutations in UQCRB, UQCRC2, and CYC1 presented with neonatal or early infancy onset, recurrent metabolic crises with elevated lactate and hypoglycaemia, from which they completely and quickly recovered with intravenous glucose. All but one showed a normal development and intellect [2, 6, 22]. A more variable clinical picture is seen with *BCS1L* mutations including lactic acidosis, renal and liver involvement, encephalopathy, hearing loss, and seizures [23]. A cohort of seven patients with *LYRM7* mutations were found to have a consistent magnetic resonance imaging pattern of progressive signal abnormalities with multifocal small cavitations in the periventricular and deep cerebral white matter [24] while another *LYRM7* patient was reported with liver involvement [10]. Patients with *TTC19* mutations are described as having an already delayed development which progresses via extrapyramidal movement disorder to a minimal residual state. One big Bedouin kindred with 25 affected individuals carrying homozygous mutations in UQCRQ has been described with a developmental delay progressing toward an extrapyramidal movement disorder, the oldest affected patients of this kindred were in their thirties [1].

Genetically, these two UQCC2 patients are distinct in terms of homozygous missense mutation in our case, while the other patient had a splice-site mutation affecting position −3 of the splice acceptor (c.214-3C>G), which might allow the formation of a small amount of wild-type protein and possibly explain the milder clinical outcome.

Biochemically, our patient presented with a severe reduction of complexes I and III of the respiratory chain in the muscle tissue. In fibroblasts, the activity of complex III was normal. This might be due to higher reserve capacity in less energy-dependent fibroblast or may be due to differences in posttranscriptional regulation as observed for complexes I and IV [25–28]. Combined deficiency of complex I and III deficiency is consistent with the previous reported patient with UQCC2 mutations who also showed reduced complex I on the level of enzymatic activity and BN-PAGE in addition to complex III deficiency. Furthermore, lack of complex III dimer and minimal complex III bound in supercomplexes in digitonin-solubilized fibroblasts of the UQCC2-deficient patient was described [7]. In agreement, we also show that in the muscle tissue of our UQCC2 patient, the formation of respiratory chain supercomplexes is diminished. Wanschers et al. reported a case with *UQCC3* mutations. Also, in this protein assembly factor disorder, combined reduction of the activity of complexes III and I was found in the muscle. In addition, diminished levels of assembled complexes III and I were present in patient fibroblasts [8]. No mutations in UQCC1 have been described so far. Both patients showed reduction of complex I and in one case complex IV activity.

In 2004, Acín-Pérez et al. observed that complex III is required to maintain complex I in mitochondria in a study with rodent cells and mutations of the cytochrome b gene, which is encoded on the mitochondrial DNA [29]. Combined deficiency of complex I and complex III was reported for some patients with *MT-CYB* mutations. In line with these findings, we and others identified homoplasmic loss of function mutations in *MT-CYB* with severe complex I and complex III deficiency in renal oncocytoma, a human tumor characterised by loss of complex I or combined deficiency of complex I and other respiratory chain enzymes [3, 30, 31]. Simultaneously, Schägger et al. also reported that primary complex III assembly deficiencies present as combined complex III/I defects also shown for cytochrome b gene mutations [32].

In *Caenorhabditis elegans*, it was shown that complex III is important for supercomplex assembly. Furthermore, it was proven that complex III uniquely affects complex I, either by decreasing the amount of complex I or the I-III-IV supercomplex. A mutation in isp-1 (ortholog of the Rieske protein) can reduce the amount of fully assembled complex I. Mutations in ctb-1 (cytochrome b ortholog) can cause a reduction in complex I activity without affecting the complex I assembly [33].

Most of the nine so far described defects of nuclear-encoded complex III subunits or assembly factors have been published after the initial and important observation in 2004. Remarkably, in most of these papers, reduction of complex I and in some cases complex IV was found in addition to complex III deficiency (Table 3):

1. Haut et al. described a boy with isolated complex III deficiency caused by a mutation in UQCRB. However, a moderate reduction of complex I activity was present in the patients' liver [22].
2. Barel et al. described a patient with UQCRQ mutation with complex III deficiency, a variable decrease of complex I activity in skeletal muscle biopsies [1].
3. Miyake et al. described a patient with a mutation in UQCRC2. The activity of complex I was increased threefold in the skeletal muscle. However, the authors showed impaired supercomplex formation in patient fibroblasts affecting complexes I and IV [6]. Very recently, Gaignard et al. also reported a patient with a reduced complex III and I activity in fibroblasts. In addition, the native complexes I and III were both reduced. Neither complex IV activity nor complex IV assembly was diminished [34].
4. Gaignard et al. reported that loss of cytochrome *c*1 encoded by *CYC1* causes an isolated complex III deficiency in two children. However, a reduced complex I activity was shown for the liver. In addition, the authors described secondary reduction of assembly-dependent subunits of complexes I and IV [2]. A reduction of supercomplexes was present as shown by BN-PAGE. Taken together, these results indicate that CYC1 mutations might cause a defect in respirasome assembly and a combined OXPHOS deficiency.
5. As reported here and in the paper of Tucker et al. [7], UQCC2 deficiency causes combined complex I and complex III deficiency. A reduction of supercomplexes was present in both cases.
6. As already mentioned above, UQCC3 deficiency causes combined complex III and complex I deficiency [8].
7. Combined deficiencies of complex III, complex I, and/or complex IV were also reported for patients with mutations in *BCS1L*, especially in patients with severe clinical symptoms [23]. In addition, BCS1L defects manifest in a highly tissue-specific pattern.
8. Two other complex III assembly factors are known LYRM7 and TTC19 although they seem to be involved in different aspects of assembly. LYRM7 plays a role in iron sulphur cluster biogenesis. However, besides severe complex III deficiency, minor reduction of complex I or complex IV in patient muscle was reported [4, 10]. The function of TTC19 is still a matter of debate although most patients show an isolated complex III deficiency. However, we reported on a TTC19 patient even without any signs of complex III changes [5].

Indeed, isolated complex III deficiency might be the exception rather than the rule. Since the presence of assembled complex III seems to be crucial for respirasome assembly and maybe for the stability or assembly of complexes I and IV. Complex III dimers are mainly found in three different complexes, one supercomplex termed respirasome (CI, CIII_2_, and CIV), one with complex I (CI, CIII_2_), or one with complex IV (CIII_2_, CIV). The supercomplexes interact at nine sites [13]. COX7a2L was the first factor designated as a supercomplex assembly factor. However, it is still debated if COX7a2L also influences respirasome assembly [35, 36]. In addition, HIGD1a was reported to promote supercomplex formation [37].

Therefore, UQCC2 deficiency, like most other disorders of complex III subunits and most assembly factors, has to be considered as disorders of respirasome assembly rather than isolated complex III defects. This finding is of diagnostic relevance since a combined reduction of complexes III and I is likely an indication for mutations in complex III-related genes. Since isolated complex III is not determined in all laboratories [38], those defects might be misdiagnosed as either complex I or complex IV deficiency. This wrong categorisation might result in selection of inappropriate panels for genetic workup.

## Figures and Tables

**Table 1:** Enzymatic activity of the OXPHOS complexes in muscle and fibroblasts.

	Muscle				Fibroblasts	
	Mean	M1	M2	Control range	Patient	Control range
Citrate synthase	140	140	139	(166–311)	210	(225–459)
Complex I	9	10	8	(30–84)	20	(18–53)
Complex I + III	30	30	30	(27–58)	152	(73–220)
Complex II	38	40	36	(53–102)	74	(64–124)
Complex II + III	34	31	36	(41–84)	125	(79–219)
Complex III	111	108	115	(230–486)	555	(208–648)
Complex IV	233	229	237	(205–739)	380	(175–403)
Complex V	79	72	85	(78–178)	92	(43–190)

Values are given in mUnits/mg protein. M1 and M2: values of two measurements.

**Table 2:** Clinical features of complex III deficiencies.

	Complex III subunits					Complex III assembly factors					
Gene	*MT-CYB*	*CYC1*	*UQCRB*	*UQCRC2*	*UQCRQ*	*UQCC2*	*UQCC2*	*UQCC3*	*TTC19*	*LYRM7*	*BCS1L*
MIM accession	516020	615453	615158	615160	615159	615824	615824	616097	615157	615838	124000
Number of patients	>50	1	1	4	25, 1 kindred	1	1, this study	1	Ca. 15	9	>30
Onset	Childhood, adulthood	Infancy, early childhood	Late infancy	Neonatal	First months of life	Intrauterine	Intrauterine	Birth	Late infancy, adulthood	Infancy, 14 years	First years, infancy
Intrauterine growth retardation						Yes	Yes				
Hearing impairment						Yes	n.a.	No			Yes
Hypotonia	Yes					Yes	Yes	Yes		Yes	Yes
Seizures						Yes	Yes	No			Yes
Abnormal EEG							Yes				Yes
Metabolic crisis			Yes					Yes		Yes	
Lactic acidosis	Yes	Yes	Yes	Yes	Yes	Yes	Yes	Yes		Yes	Yes
Increased CSF lactate	Yes					Yes					
Hypoglycaemia		Yes	Yes	Yes				Yes			
Developmental disability		No	No	No		No	n.a.	Yes	Yes	Yes	Yes
Intellectual disability		No	No	Yes (1)/no (1)	Yes		n.a.	Yes		Yes	Yes
Other features		In one hyperammonemic liver failure			Extrapyramidal movement disorder, survival into thirties	Renal tubular acidosis, no information after 9 years of age	Status epilepticus, died at 33 days of life	Muscular weakness	Later regression with spasticity and movement disorder leading to minimal residual state	Deterioration after metabolic crises, specific MRI pattern (multifocal cavitating leukoencephalopathy)	Hepatopathy, renal involvement, often early death

**Table 3:** Results of enzymatic investigations and BN-PAGE electrophoresis of patients with complex III deficiencies.

Reduction of enzymes [tissue]	MT-CYB	CYC1	UQCRB	UQCRC2	UQCRQ	UQCC2	UQCC2	UQCC3	TTC19	LYRM7	BCS1L
Complex III [muscle]	Yes	3§/3	n.a.	1§/1	3/3 (22 = n.a.)	1/1	1/1	1/1	Most	6/9 (3 = n.a.)	Reduced
Complex III [fibroblasts]	Yes	2/2	1/1	2/2 (2 = n.a.)	n.a.	1/1	0/1	1/1	Some	1/9 (8 = n.a.)	Reduced
Complex III [liver]		1/1 (1 = n.a.)	1/1	n.a.	n.a.	n.a.	n.a.	n.a.	n.a.	1/9 (8 = n.a.)	Variable
Complex I [muscle]	Yes	1§/2 (1 = n.a.)	n.a.	1§/1	3/3 (22 = n.a.)	1/1	1/1	1/1	Normal	1/9 (8 = n.a.)	Variabe
Complex I [fibroblasts]		n.a.	n.a.	1/2 (2 = n.a.)	n.a.	1/1	0/1	0/1	Normal	n.a.	Variable
Complex I [liver]		1/1 (1 = n.a.)	1/1	n.a.	n.a.	n.a.	n.a.	n.a.	n.a.	n.a.	Variable
Complex I [BN-PAGE] fibroblasts	Muscle	1/2	n.a.	2/2 (2 = n.a.)	n.a.	1/1	0/1	1/1	Normal	0/1 (8 = n.a.)	Variable
Complex IV [muscle]		1§/3	n.a.	1§/1	2/3 (22 = n.a.)	1/1	Reduced	0/1	Normal	n.a.	Variable
Complex IV [fibroblasts]		0/1 (1 = n.a.)	1/1	0/2 (2 = n.a.)	n.a.	1/1	0/1	0/1	Normal	n.a.	Variable
Complex IV [liver]		0/1 (1 = n.a.)	0/1	n.a.	n.a.	n.a.	n.a.	n.a.	n.a.	n.a.	
Complex IV [BN-PAGE] fibroblasts		0/2	n.a.	1/2 (2 = n.a.)	n.a.	Normal	n.a.	0/1	Normal	0/1 (8 = n.a.)	Variable
Literature	Gasparre et al. [3] Zimmermann et al. [9] Blakely et al. [39]	Gaignard et al. [34], §one patient from Tucker et al. [7], no values provided	Haut et al. [22]	Gaignard et al. [34], Gasparre et al. [3], §one patient from Tucker et al. [7], no values provided	Barel et al. [1]	Tucker et al. [7]	This study	Wanschers et al. [8]	Koch et al. [5], Ardissone et al.	Invernizzi et al. [4] Dallabona et al. [24] Kremer et al. [10]	Moran et al. [23]

## Supplementary Materials

Supplementary Materials Supplementary table 1: Prediction of pathogenicity.
