# Supplementary material for: Combined Respiratory Chain Deficiency and UQCC2 Mutations in Neonatal Encephalomyopathy: Defective Supercomplex Assembly in Complex III Deficiencies
Source: Oxid Med Cell Longev. 2017 Jul 19;2017:7202589. doi: 10.1155/2017/7202589 (PMC5540226; doi:10.1155/2017/7202589)
Supplement: Supplementary Materials — Supplementary table 1: Prediction of pathogenicity. [file 7202589.f1.pdf]

Supplementary table 1: Prediction of pathogenicity

**Mutation**	**c.23G>C**	**c.28C>T**
Chromosomal location	chr6:33679441C>G	chr6:33679436G>A
Protein change	p.Arg8Pro	p.Leu10Phe
Polyphen-2 score (max. 1)	0.991	0.934
Polyphen-2 prediction	Probably damaging	Possibly damaging
SIFT score (cutoff<0.050)	0.034	0.009
SIFT prediction	Damaging	Damaging
Provean score (cutoff<-2.5)	-4.33	-2.88
Provean prediction	Deleterious	Deleterious
MutationTaster score (max. 1)	0.999997704040623	0.996059894177104
MutationTaster prediction	Disease causing	Disease causing
CADD score	25.2	27.3
